# Supplementary material for: Priority age targets for COVID-19 vaccination in Ethiopia under limited vaccine supply
Source: Sci Rep. 2023 Apr 5;13:5586. doi: 10.1038/s41598-023-32501-y (PMC10075159; doi:10.1038/s41598-023-32501-y)
Supplement: Supplementary file 1 — Supplementary Information. [file 41598_2023_32501_MOESM1_ESM.pdf]

Supplementary Material

Priority age targets for COVID-19 vaccination in Ethiopia under limited vaccine supply

Margherita Galli[1,2,#], Agnese Zardini[1,#], Worku Nigussa Gamshie[3], Stefano Santini[4], Ademe Tsegaye[5], Filippo Trentini[1,6], Valentina Marziano[1], Giorgio Guzzetta[1,7], Mattia Manica[1,7], Valeria d'Andrea[1], Giovanni Putoto[4], Fabio Manenti[4], Marco Ajelli[8,§], Piero Poletti[1,7,§,*], Stefano Merler[1,7,§]

[#] equally contributed.
[§] these authors contributed equally as senior authors.
* corresponding author: poletti@fbk.eu

[1] Center for Health Emergencies, Bruno Kessler Foundation, Trento, Italy
[2] Department of Mathematics, Computer Science and Physics, University of Udine, Udine, Italy
[3] Doctors with Africa CUAMM, Woliso, Ethiopia
[4] Doctors with Africa CUAMM, Padova, Italy
[5] Doctors with Africa CUAMM, Addis Abeba, Ethiopia
[6] Dondena Centre for Research on Social Dynamics and Public Policy, Bocconi University, Milan, Italy
[7] Epilab-JRU, FEM-FBK Joint Research Unit, Trento, Italy
[8] Laboratory for Computational Epidemiology and Public Health, Department of Epidemiology and Biostatistics, Indiana University School of Public Health, Bloomington, IN, USA

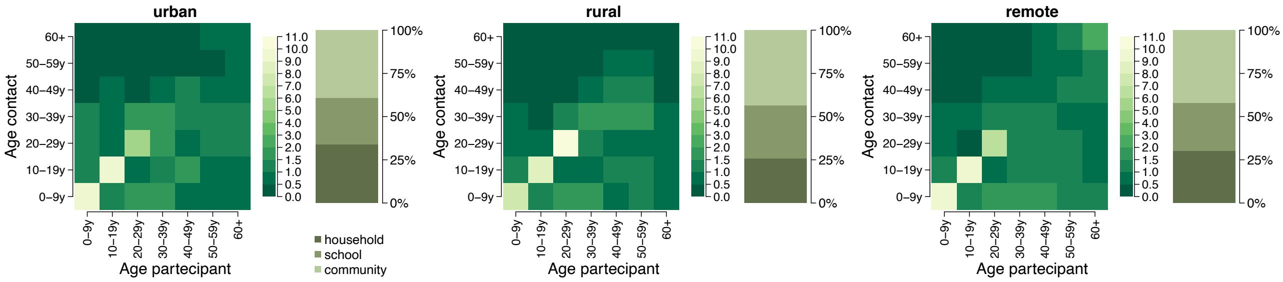

Figure S1. Contact matrices representing the mean number of daily contacts reported by a participant in the age group i with individuals in the age group j in each site (urban, rural, and remote). The bar plots show the percentage of contacts that occurred in each setting (household, school, and community).

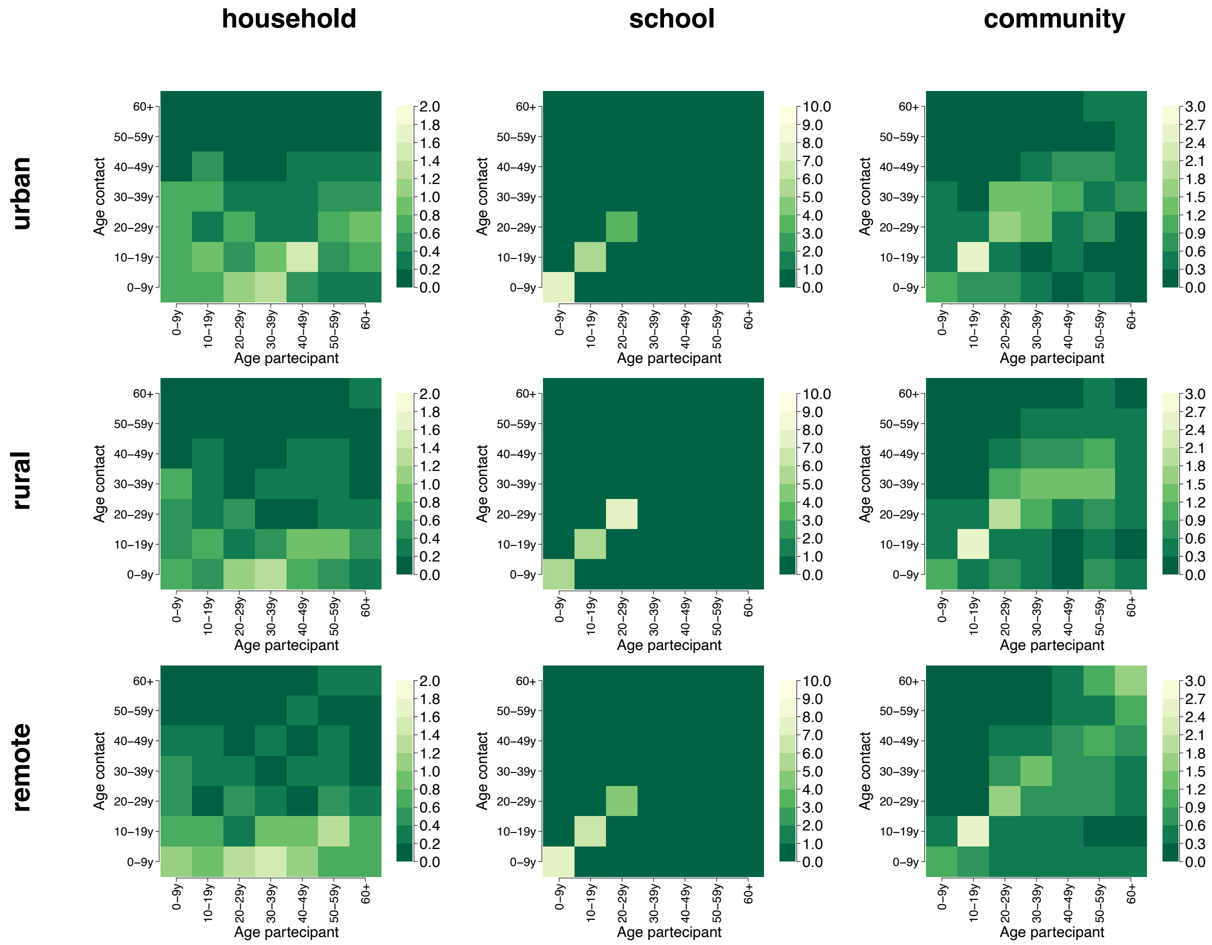

Figure S2. Contact matrices representing the mean number of daily contacts reported by a participant in the age group i with individuals in the age group j in each setting (household, school, and community) and site (urban, rural, and remote).

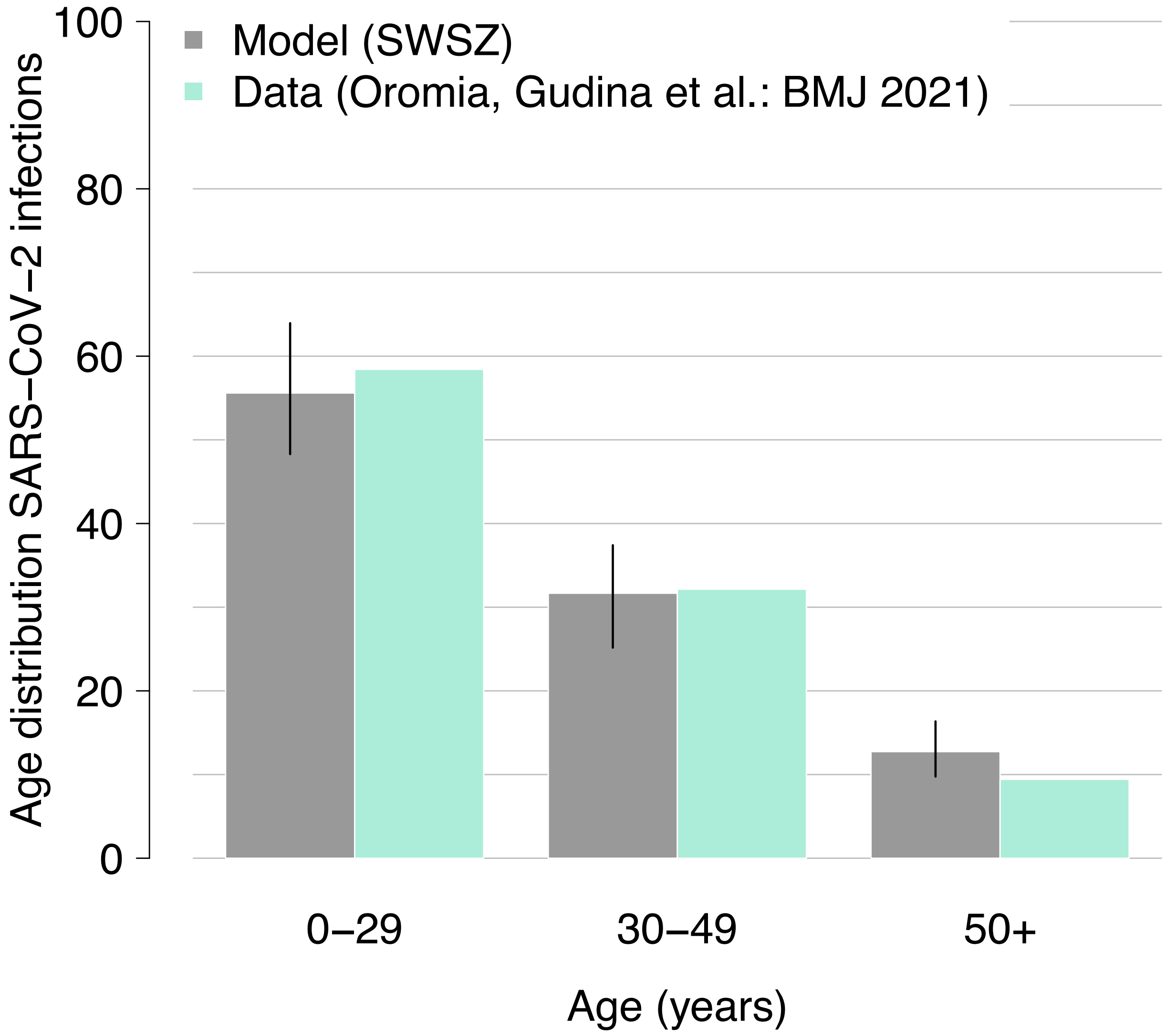

Figure S3. Comparison between the age distribution of all confirmed cases reported between March and September 2020 in the Oromia Region [1] and the age distribution of the cumulative infections as obtained with a model mimicking the school closure and the achievement of immunity levels estimated for the Jimma Zone in December 2020 [2]. Aggregated age distribution for the entire SWSZ was obtained by summing up the estimated number of cases across remote settlements, rural villages, and in urban neighborhoods of the SWSZ for each age class [3].

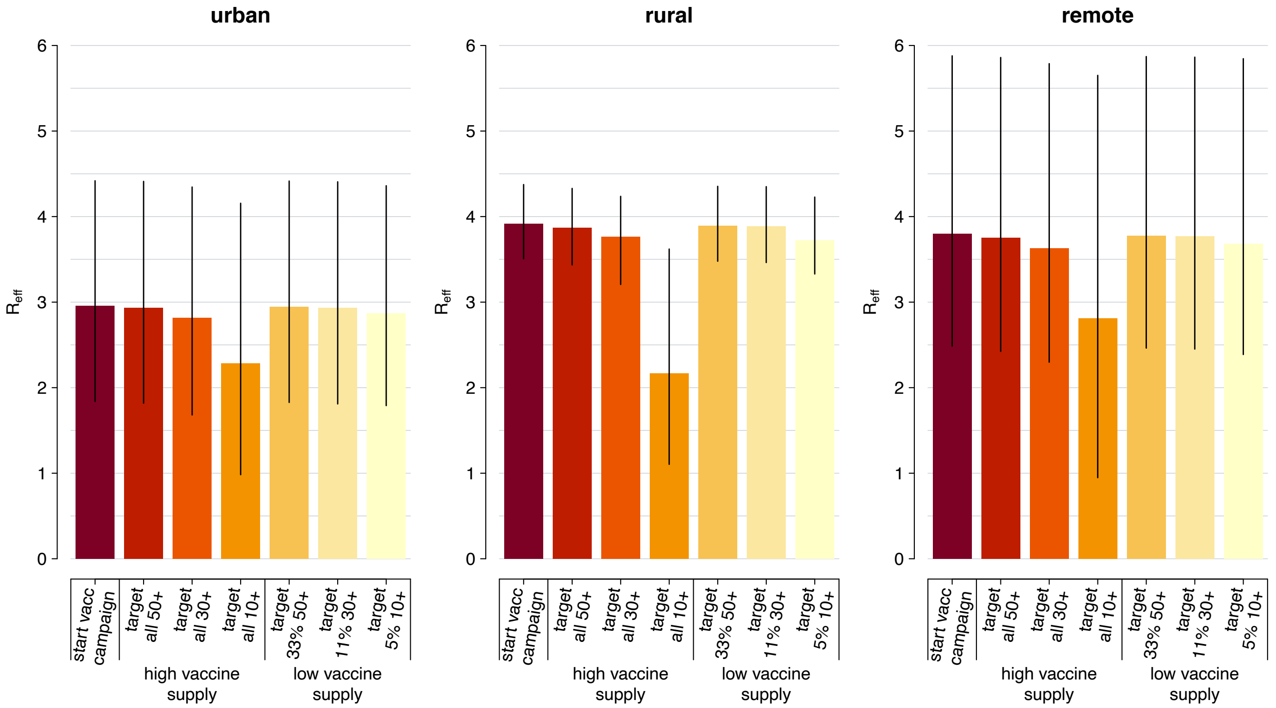

Figure S4. Estimated effective reproduction number R_{eff} at the launch of the vaccination campaign and under different vaccination strategies. In the scenario of low vaccine supply, we considered a 3.4% coverage in the population (reached in Ethiopia at the end of 2021 [4]) and we analyzed the impact of uniformly administering the same number of vaccine doses to individuals aged more than 50, 30, or 10 years.

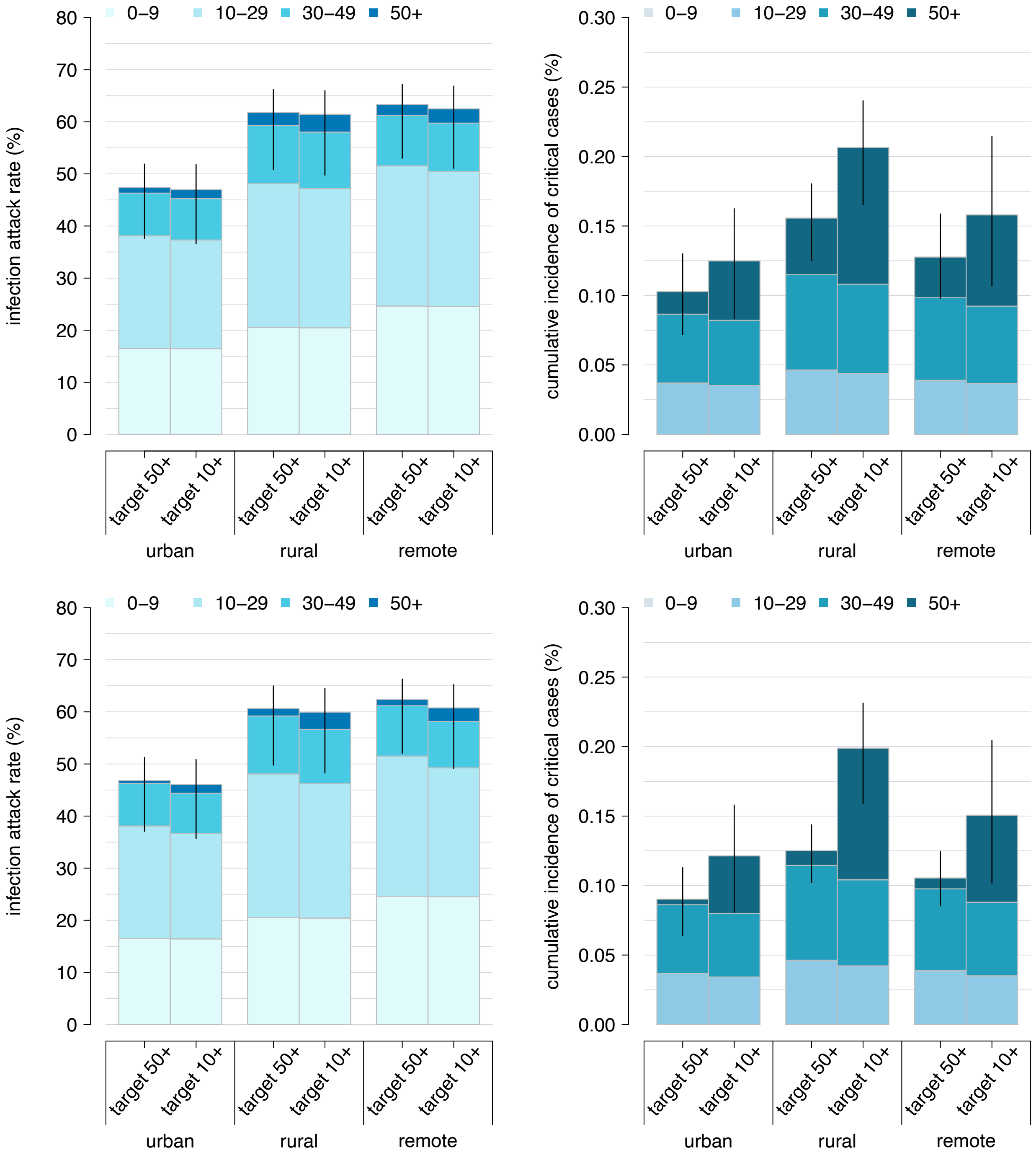

Figure S5. Sensitivity analysis on vaccine efficacy. Estimated infection attack rate and cumulative incidence of critical cases expected across different geographical contexts (urban, rural, and remote), as obtained under the assumption that either all the individuals aged 50 years or older are vaccinated or the corresponding number of vaccine doses is uniformly distributed throughout the population over 10 years. Estimates are obtained assuming a lower vaccine efficacy (set at 55% against infection and 45% against critical disease; first row) and a more effective vaccine (with an efficacy of 80% against infection and 75% against critical disease; second row). Colored bars represent average estimates, stratified by the age group of infected individuals (0-9, 10-29, 30-49, 50+ years); solid lines represent the 95% PI of model estimates.

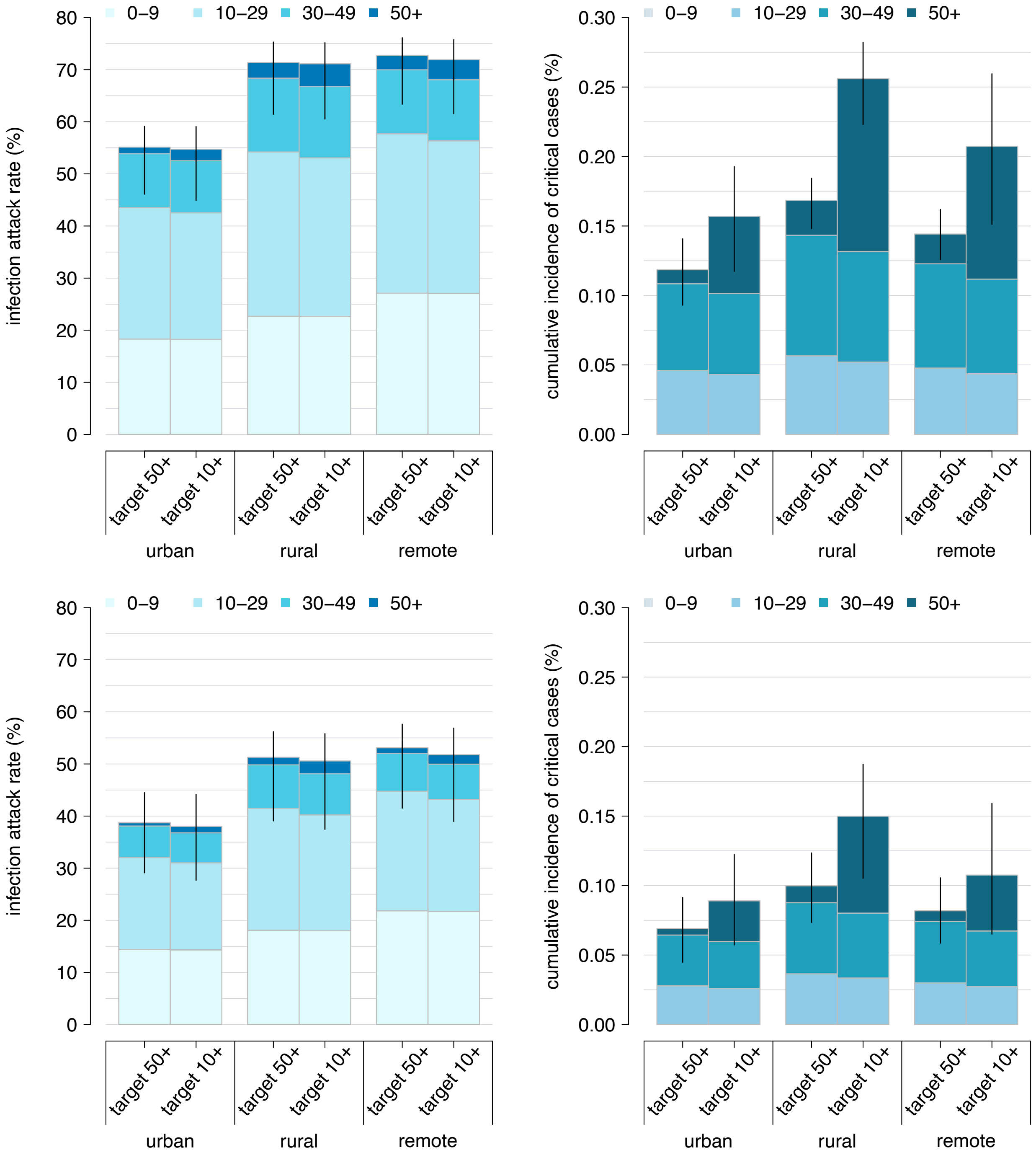

Figure S6. Sensitivity analysis on initial natural immunity. Estimated infection attack rates and cumulative incidence of critical cases expected across different geographical contexts (urban, rural, and remote), as obtained under the assumption that either all the individuals aged 50 years or older are vaccinated or the corresponding number of vaccine doses is uniformly distributed throughout the population over 10 years. Estimates are obtained assuming a lower initial natural immunity (22% in rural and in remote, 38% in urban; first row) and higher initial immunity levels (40% in rural and in remote, 53% in urban; second row). Colored bars represent average estimates, stratified by the age group of infected individuals (0-9, 10-29, 30-49, 50+ years); solid lines represent the 95% PI of model estimates.

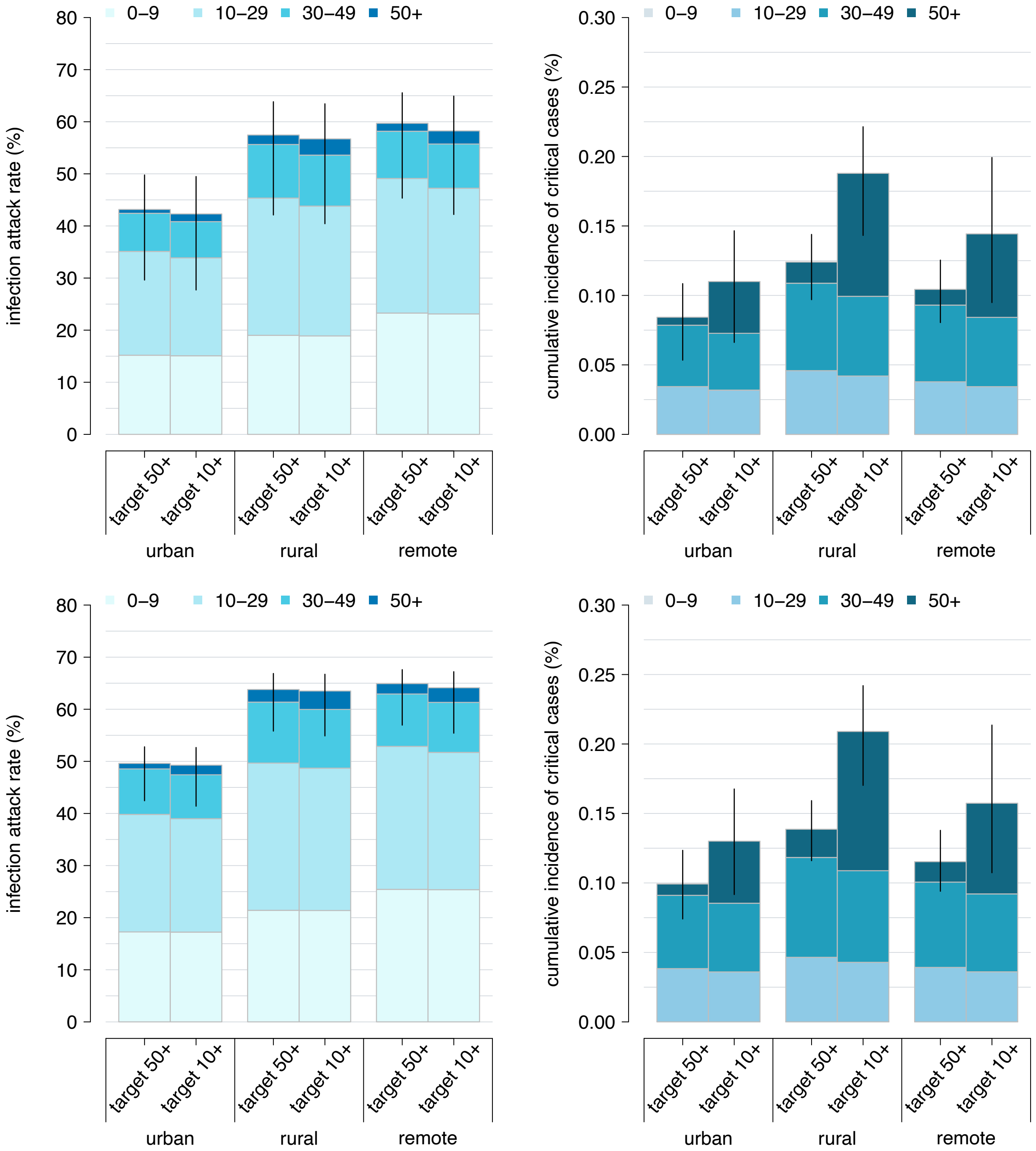

Figure S7. Sensitivity analysis on transmissibility. Estimated infection attack rates and cumulative incidence of critical cases expected across different geographical contexts (urban, rural, and remote), as obtained under the assumption that either all the individuals aged 50 years or older are vaccinated or the corresponding number of vaccine doses is uniformly distributed throughout the population over 10 years. Estimates are obtained assuming a 15% decrease (first row) and a 15% increase (second row) in the SARS-CoV-2 transmissibility. Colored bars represent average estimates, stratified by the age group of infected individuals (0-9, 10-29, 30-49, 50+ years); solid lines represent the 95% PI of model estimates.

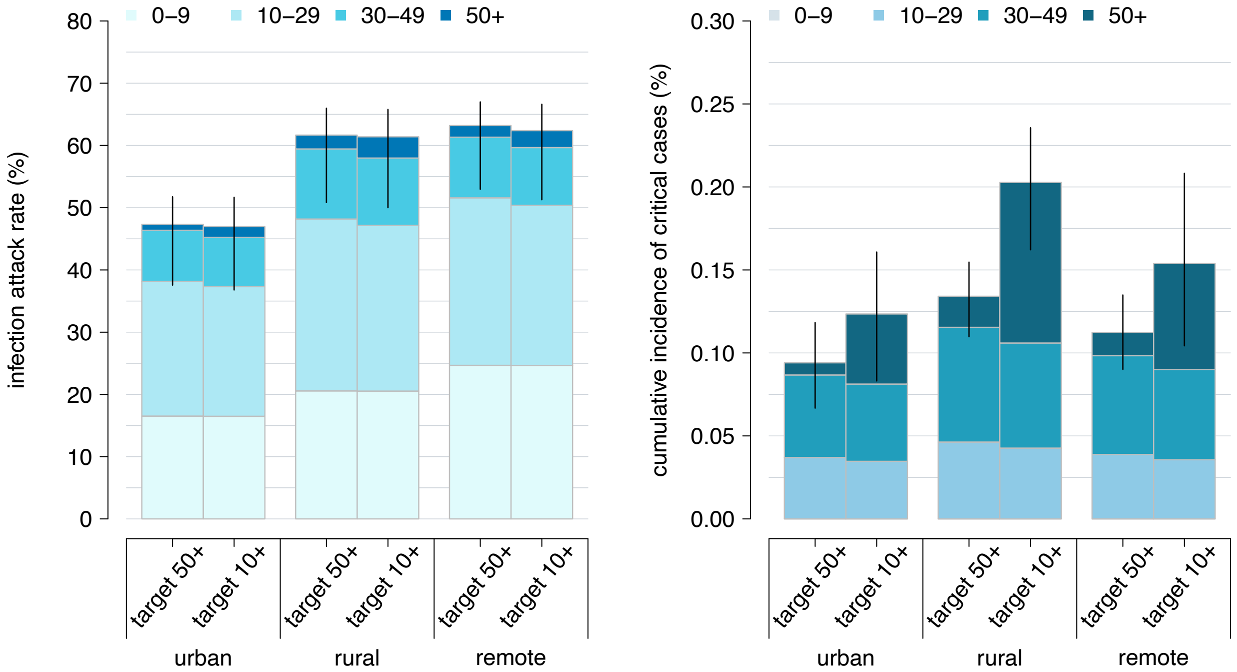

Figure S8. Sensitivity analysis on infectiousness of vaccinated and unvaccinated infections. Estimated infection attack rates and cumulative incidence of critical cases expected across different geographical contexts (urban, rural, and remote), as obtained under the assumption that either all the individuals aged 50 years or older are vaccinated or the corresponding number of vaccine doses is uniformly distributed throughout the population over 10 years. Estimates are obtained assuming a homogeneous infectiousness of breakthrough infections and infections among unvaccinated individuals. Colored bars represent average estimates, stratified by the age group of infected individuals (0-9, 10-29, 30-49, 50+ years); solid lines represent the 95% PI of model estimates.

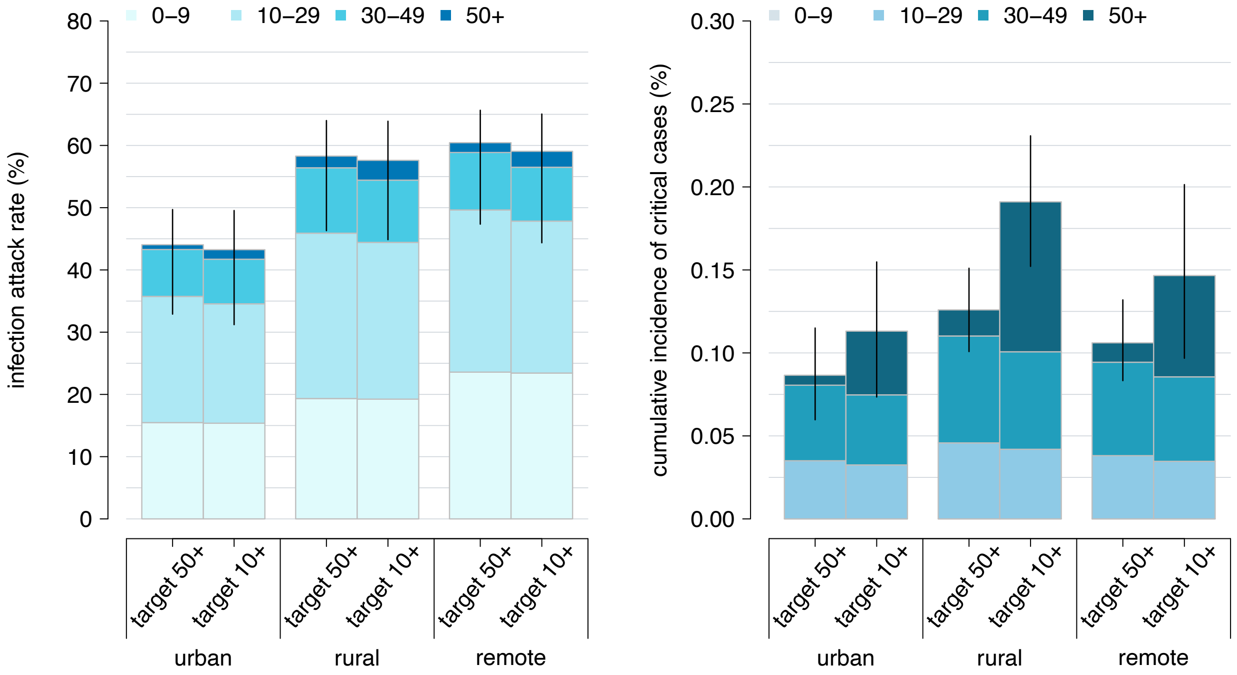

Figure S9. Sensitivity analysis on susceptibility. Estimated infection attack rates and cumulative incidence of critical cases expected across different geographical contexts (urban, rural, and remote), as obtained under the assumption that either all the individuals aged 50 years or older are vaccinated or the corresponding number of vaccine doses is uniformly distributed throughout the population over 10 years. Estimates are obtained assuming a homogeneous susceptibility by age during the Delta wave. Colored bars represent average estimates, stratified by the age group of infected individuals (0-9, 10-29, 30-49, 50+ years); solid lines represent the 95% PI of model estimates.

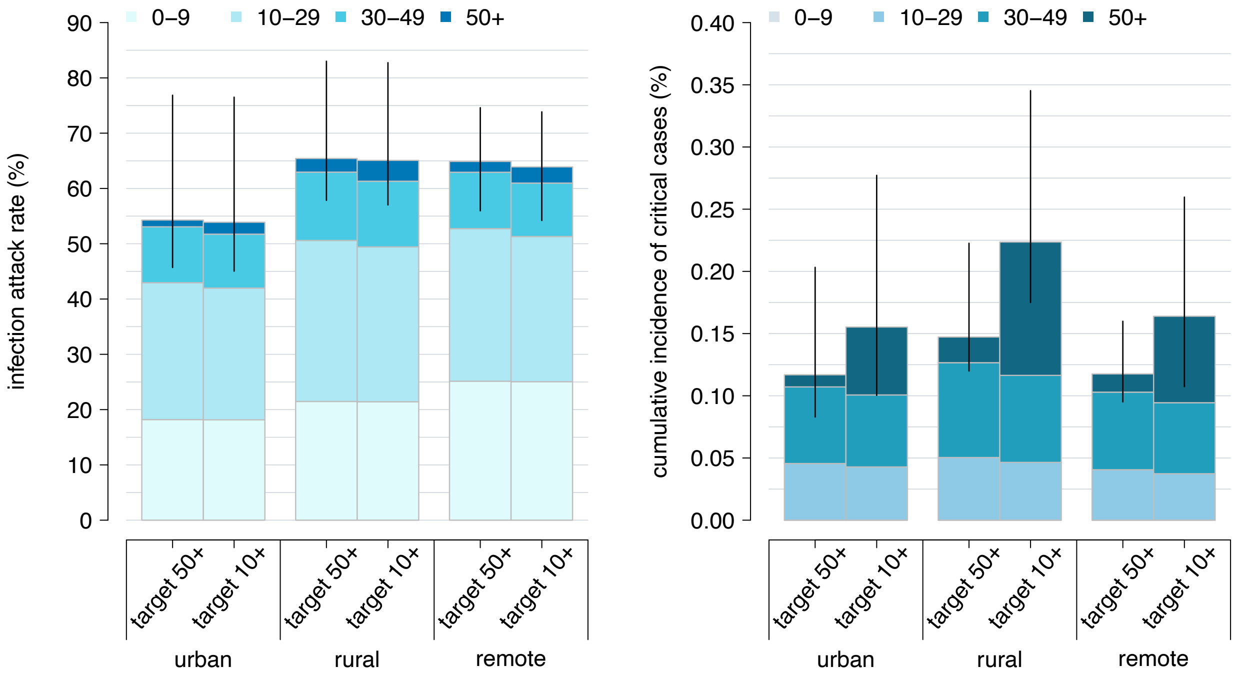

Figure S10. Sensitivity analysis on transmissibility of ancestral strains. Estimated infection attack rates and cumulative incidence of critical cases expected across different geographical contexts (urban, rural, and remote), as obtained under the assumption that either all the individuals aged 50 years or older are vaccinated or the corresponding number of vaccine doses is uniformly distributed throughout the population over 10 years. Estimates are obtained assuming R_0 = 2.55 for the ancestral strain, as estimated by Iyaniwura et al. [5]. Colored bars represent average estimates, stratified by the age group of infected individuals (0-9, 10-29, 30-49, 50+ years); solid lines represent the 95% PI of model estimates.

References

1. Gudina EK, Gobena D, Debela T, Yilma D, Girma T, Mekonnen Z, et al. COVID-19 in Oromia Region of Ethiopia: a review of the first 6 months' surveillance data. BMJ Open 2021;11:e046764.

2. Gudina EK, Ali S, Girma E, Gize A, Tegene B, Hundie GB, et al. Seroepidemiology and model-based prediction of SARS-CoV-2 in Ethiopia: longitudinal cohort study among front-line hospital workers and communities. Lancet Glob. Health 2021;9:e1517–27.

3. Trentini F, Guzzetta G, Galli M, Zardini A, Manenti F, Putoto G, et al. Modeling the interplay between demography, social contact patterns, and SARS-CoV-2 transmission in the South West Shewa Zone of Oromia Region, Ethiopia. BMC Med. 2021;19:89.

4. Ethiopian Public Health Institute. COVID-19 PANDEMIC PREPAREDNESS AND RESPONSE IN ETHIOPIA, WEEKLY BULLETIN Epi-Week- 51 (December 20 – 26, 2021). [Internet]. 2022 [cited 2022 Apr 6];Available from: https://ephi.gov.et/wp-content/uploads/2021/02/EPHI_PHEOC_COVID-19_Weekly_Bulletin_87_English_01032021.pdf

5. Iyaniwura SA, Rabiu M, David JF, Kong JD. The basic reproduction number of COVID-19 across Africa. PloS One 2022;17:e0264455.
